# A Survey of New South Wales Sheep Producer Practices and Perceptions on Lamb Mortality and Ewe Supplementation

**DOI:** 10.3390/ani10091586

**Published:** 2020-09-05

**Authors:** Kayla Kopp, Marta Hernandez-Jover, Susan Robertson, Angel Abuelo, Michael Friend

**Affiliations:** 1 Graham Centre for Agricultural Innovation (NSW Department of Primary Industries and Charles Sturt University), Albert Pugsley Place, Wagga Wagga, NSW 2650, Australia; mhernandez-jover@csu.edu.au (M.H.-J.); surobertson@csu.edu.au (S.R.); 2 School of Animal and Veterinary Sciences, Charles Sturt University, Locked Bag 588, Wagga Wagga, NSW 2678, Australia; 3 College of Veterinary Medicine, Michigan State University, 784 Wilson Road, East Lansing, MI 48824, USA; abuelo@msu.edu; 4 Office of the Pro-Vice Chancellor, Research and Innovation, Charles Sturt University, Locked Bag 588, Wagga Wagga, NSW 2678, Australia; mfriend@csu.edu.au

**Keywords:** sheep production, lambing, mortality, starvation, predation, mis mothering, supplementation

## Abstract

**Simple Summary:**

High lamb mortality rates following birth reduce on-farm profitability and contribute to perceived lower animal welfare standards of the sheep industry. The aim of this study was to understand producer knowledge of lamb mortality rates, causes of lamb mortality, and to investigate practices and perceptions of producers that may contribute to lamb deaths. Approximately 50% of producers estimated less than 10% lamb mortality between birth and marking, compared to published data in Australia reporting around 20–25% mortality rate. Clostridial vaccination of lambs was undertaken by 96% of producers; however, 17% of Merino and 23% of crossbred lamb producers indicated only one vaccination was administered, instead of the recommended initial vaccination plus booster. This lower estimated mortality rate and misuse of vaccination may lead to producers underestimating the perceived benefits of management strategies, as the number of lambs lost is of less concern. It is important producers are aware of the actual on-farm lamb losses to allow accurate determination of the benefits of management strategies, such as pre-lambing supplementation and vaccination.

**Abstract:**

High lamb mortality rates reduce profitability and reduce the perceived animal welfare standards of the industry. This study aimed to understand producer knowledge of lamb mortality rates and causes of lamb mortality, and to investigate various practices and perceptions of producers that may contribute to lamb deaths. Postal and online surveys gathered data on Australian sheep producer’s knowledge and practices around lambing and management practices. Based on results, approximately 50% of producers estimated less than 10% mortality of lambs between birth and marking, compared to published data estimates of 20–25% mortality. Pre-lambing vaccination of ewes was not undertaken by 10–20% of producers. Ninety-six percent of producers vaccinated lambs; however, 17% of Merino and 23% of crossbred lamb producers only gave a single vaccination instead of the recommended initial vaccine and booster. The lower estimated mortality impacts producer’s perceived benefits of management strategies being undertaken. Research undertaken needs to be more effectively distributed to producers via extension services to ensure producers understand the causes of mortality. Important messages to convey to producers include the limited impact of predation in most cases and the total costs of lamb mortality on-farm.

## 1. Introduction

Mortality rates of lambs from birth to weaning continue to be a major source of lost production reducing profitability in sheep production systems [1]. Average lamb mortality rates of 10% for single-born lambs and 30% for twin-born lambs are common in Australia, but mortality can be up to 70% [1], leading to a perception of low animal welfare standards. 

As more than 10 million lambs die before weaning in Australia [2], the causes of lamb mortality are of major importance. Dystocia, starvation, and exposure have been identified as the major causes of lamb mortality [3]. As the major causes of mortality are affected by birthweight, with larger lambs likely to be affected by dystocia [4] with neonatal lamb growth and lactation affected by nutritional status [5], representing an area producers can alter management practices to increase survival. With perinatal lamb mortality rates recognized as a significant issue affecting sheep producers, research has concentrated on methods to increase lamb survival, with a focus on management practices, including supplementation.

Pre-lambing supplementation of ewes with cereal grains, such as maize has increased colostrum production [6], which would be likely to impact lamb survival through increased nutrition for growth, immunity, and heat production. Supplementation of ewes with calcium and magnesium has been found to increase ewe weight gains with calcium, and lamb weight gains with magnesium [7,8]. Calcium and magnesium are involved in milk production and smooth muscle contraction for parturition. As a result, the increased weight gains may also have some benefits to lamb survival. Therefore, exploring whether producers have implemented these supplementation practices may provide further understanding of the benefits of supplementation. Supplementation of ewes in late pregnancy would belikely to improve lamb survival [9] through increasing birthweight [10] and colostrum/milk production [5,6,11]. Management practices of scanning ewes and lambing twins and singles separately to manage nutritional requirements has increased twin lamb survival [12]. The practice of vaccinating ewes is also beneficial [13,14] to reduce the mortality rates and increase production.

As animal welfare issues continue to be of great concern in the industry, and there are known practices that improve survival, it is important to understand producer knowledge and uptake of these practices. Published data are not available on the extent to which NSW producers are using existing options to reduce the mortality rates on-farm, and if farmers are aware of the number of lambs lost from birth to marking. Therefore, the objectives of this study were to understand producer perceptions on lamb mortality and the various practices producers implement to reduce post-natal lamb mortality rates on-farm.

## 2. Materials and Methods

### 2.1. Recruitment and Questionnaire

A 30-question survey on management practices of ewes and lambs and mortality around lambing was distributed to sheep producers across the state of NSW (Australia) between May and October 2019. Initially, a pilot survey was distributed to three producers, with their feedback used to revise the survey prior to wider circulation. These responses were not included in the final dataset as the questions were revised. The survey was made available online via SurveyMonkey, and paper copies were offered to producers at a producer meeting. As a result of the online distribution method the percentage of respondents is unknown. To assist with distribution a variety of organizations/businesses within the sheep industry circulated the online link to members or readers. The sample size required was estimated using the Epitools calculator (epitools.ausvet.com.au), to estimate a simple proportion assuming an estimated 40–50% proportion of producers conducting a specific practice, 10% desired precision of the estimate, a 95% confidence level and infinite population (>1000). According to these assumptions, the required sample size was just under 100 (93 to 97).

To participate in the survey producers must have been either farm owners or managers, had ewes lambing on the property in 2019 and had more than 50 sheep on the property. The survey was kept short to maximize the number of respondents. Survey questions focused on practices and perceptions of sheep producers around scanning, supplementation at lambing, and vaccination practices and producers identified risk factors associated with lamb mortality. Twenty-three close-ended questions and seven open-ended questions were asked. Close-ended questions were used to identify postcode, age, years farming, generations farming, causes of lamb mortality, and reasoning for supplementary feeding. Close-ended questions were also asked to identify gender, farming system, number of ewe breeds, lambing time, vaccination, scanning, marking percentages, when supplementary feed was provided, amount of supplement, and reasoning for not providing a cost-effective supplement. For further details, the survey is available in the Supplementary Materials. The survey was approved by the Charles Sturt University Human Research Ethics Committee (Approval No: H19061).

Marking of lambs in Australian flocks is generally undertaken at 2–8 weeks of age, following the end of the lambing period. At marking, husbandry procedures likely to be undertaken include tail docking, ear tagging, ear marking, vaccination, and castration of male lambs. Weaning in Australia is recommended around 14 weeks following the start of the lambing period (lambs aged 8–14 weeks at weaning) [15]. At weaning, it is recommended lambs receive the second clostridial vaccination.

### 2.2. Statistical Analysis

Responses were entered into Microsoft Excel [16] and exported into SPSS [17] for statistical analysis. Data were cleaned before exporting into SPSS. All variables were examined with descriptive statistics to determine their distributions. Each categorical variable was examined using frequencies. The associations between management practices (farm system, lambing time, vaccination, scanning and supplementation) and estimated mortality rates of lambs between birth to marking and marking to weaning were examined using ordinal logistic regression via plum analysis with the model considered to be significant if the likelihood ratio test *p*-value < 0.05.

## 3. Results

**Figure 1:**
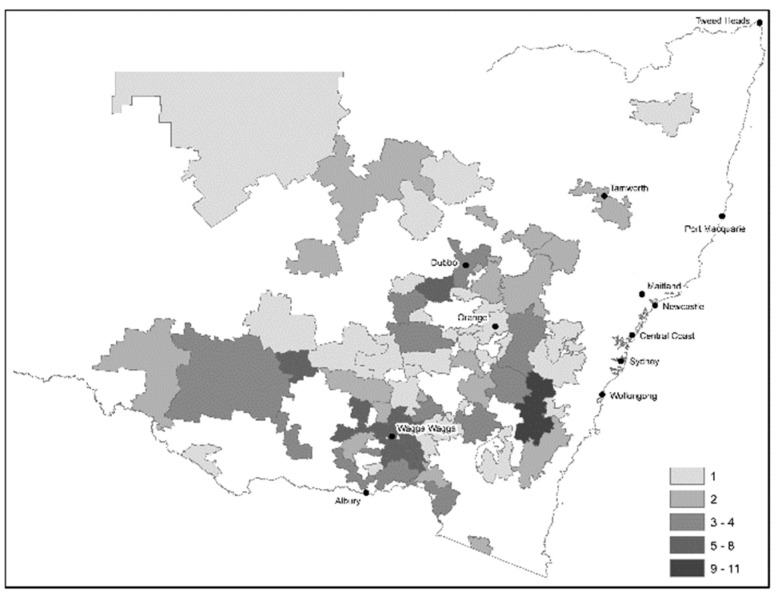
New South Wales map of distribution of the 2019 sheep survey participants *(n* = 145) by postcode. Average annual daily temperatures throughout New South Wales vary from minimum −1.9 °C to maximum 36.3 °C.

A total of 178 producers participated in the survey, although 33 responses were unable to be used due to producers either not being located in NSW, duplicate response, having less than 50 sheep, or only answering on population demographics. Usable survey respondents included 145 NSW-based sheep producers. Of these producers, 23% were sheep-only producers, 24% produced sheep and cattle, 25% were sheep, cattle, and cropping farmers, while 28% were sheep and cropping farmers. Producers varied in age from 18 to 78 years with a median of 44 years (Interquartile Range (IQR) 31–55 years). These producers had been farming for between 1 and 65 years with a median of 25 years (IQR 10–37 years). Participating producers were from 1st to 7th generation farmers, with a median of 4 generations. The location of producers completing the survey is shown in Figure 1.

### 3.1. Sheep Demographics

Producers participating identified all ewe breeds on-farm with some producers having several breeds of ewe. Purebred Merino ewes were owned by 75% (*n* = 107) of producers, with median ewe numbers of 1250, ranging from 75 to 20,000. Meat breed ewes were owned by 14% (*n* = 21) of participating producers with a median mob size of 270 ranging from 36 to 3000 breeding ewes. Purebred meat breeds included Australian Whites, Border Leicester, Dohne, Dorper, Poll Dorset, South African Meat Merino, Suffolk, White Suffolk, and Wiltipoll. Crossbred ewes were owned by 35% (*n* = 51) of participating producers with a median mob size of 850 ranging from 20 to 10,000 breeding ewes. Lamb breed was also identified with 65% (*n* = 89) of producers breeding purebred Merino lambs and 72% (*n* = 97) breeding purebred meat or crossbred lambs.

### 3.2. Reproductive Management

In Australia, there are two main lambing periods, autumn lambing from February to May and winter/spring lambing from June to September, with some producers using a continuous breeding cycle where rams remain with ewes year-round. Few producers use the three-lambings-every-two-years management practice, usually in meat operations to maximize ewe production. Lambing times indicated by producers are summarized in Table 1. Among participating producers, scanning of ewes pre-lambing was completed by 82% (*n* = 78) of Merino ewe owners, 71% (*n* = 14) of purebred meat ewe owners and 76% *(n* = 38) of crossbred ewe owners. The distribution and type of scanning practice identified by those producers scanning ewes are identified in Table 2.

### 3.3. Animal Health Management

Vaccination of ewes pre-lambing was reported by most producers. Among participating producers, 79% (*n* = 84) vaccinated Merino ewes, 90% (*n* = 19) vaccinated meat breed ewes and 80% (*n* = 41) vaccinated crossbred ewes. The types of vaccinations administered to ewes are detailed in Table 3.

Producers were also asked about the reasons they vaccinate ewes. Seventy-nine percent (*n* = 88) of producers vaccinated to increase immunity transfer to lambs, while 74% (*n* = 82) vaccinated to increase lamb survival rates. Fifty-five percent (*n* = 61) of producers chose to vaccinate because they had always vaccinated. Producers who vaccinated also sought advice about vaccination from veterinarians 13% (*n* = 14), animal nutritionists 10% (*n* = 11), family members 8% (*n* = 9) and rural stores 8% (*n* = 9). Fewer producers (*n* =5) chose to vaccinate ewes due to education course participation, understanding technical data identifying benefits, keeping the ewe healthy, increasing ewe survival, and participating in trial protocols.

Among producers who chose not to vaccinate, reasoning included time constraints (59%, *n* = 17), cost (31%; *n* = 9), and the thought that the vaccination was ineffective (21%; *n* = 6). Other reasons identified (*n* = 3 producers) included having never vaccinated, never previously had issues with disease and the belief there is a lack of research around vaccine effectiveness.

Vaccination of lambs was undertaken by most producers (96%) with four Merino lamb and four crossbred lamb producers not vaccinating. Table 4 shows the vaccinations used by producers. The majority of producers vaccinated at marking and weaning (Merino *n* = 68 (83%); crossbred *n* = 72 (77%)), with some producers vaccinating at marking only (Merino *n* = 12 (15%); crossbred *n* = 21 (23%)) or at weaning only (Merino *n* = 1 (2%)). Producers who did not vaccinate did so as they either identified as organic farmers, thought vaccination was not cost-effective, too costly, time constraints, or did not identify a reason.

Among producers vaccinating Merino lambs, 69% (*n* = 59) vaccinated to increase immunity levels, 65% (*n* = 58) to increase lamb survival and 47% (*n* = 42) vaccinated because they have always vaccinated. Producers who vaccinated also sought guidance from veterinarians 12% (*n* = 11), rural stores 7% (*n* = 6), animal nutritionists 7% (*n* = 6), and family members 3% (*n* = 3). Additional explanations identified by three producers for vaccination included for carcass characteristics, previous pulpy kidney issues, and adherence to trial protocols.

Among the respondents vaccinating crossbred lambs, 69% (*n* = 67) vaccinated to increase immunity levels, 62% (*n* = 60) to increase lamb survival, and 62% (*n* = 60) vaccinated because they have always vaccinated. Producers who vaccinated crossbred lambs also sought advice from veterinarians 13% (*n* = 13), rural stores 10% (*n* = 10), animal nutritionists 6% (*n* = 7) and from family members 4% (*n* = 4). A single producer indicated previously having Ovine Johne’s Disease as the reason for vaccination.

### 3.4. Lamb Survival and Lamb Mortality

Lamb marking rates reported by producers from birth to marking, are shown in Table 5. Lamb marking rates for all ewe types (Merino, crossbred and meat) were identified by producers to be higher in good years with high feed availability, compared to bad years with low feed availability. Meat and crossbred ewes had higher producer-reported lamb marking percentages in good and bad years compared to Merino ewes. Producers were asked to identify the causes of lamb mortality within their flock by ewe breed. Producers estimated, via percentages, the causes of mortality adding up to 100%. Producers reported the factors they believed contribute to lamb mortality by ewe breed, which are presented in Table 6. Producers estimated the starvation–mismothering–exposure complex to be the major cause of mortality in all ewe breeds (40–49% of lamb deaths), with predation and dystocia estimated to cause similar levels of mortality (18–21% of lamb deaths). A wide variation in producer estimations on the causes of lamb mortality were found.

**Figure 2:**
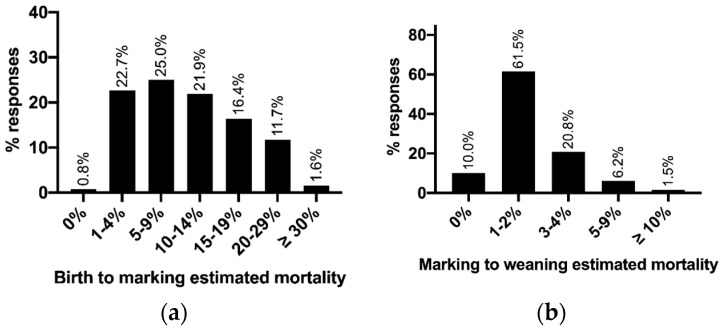
Producer estimated lamb mortality rates from a 2019 NSW sheep producer survey: (**a**) from birth until marking (*n* = 128); (**b**) from marking to weaning (*n* = 130).

Forty-nine percent of producers estimated mortality of lambs between birth and marking to be 9% or less, as shown in Figure 2a. Mortality rates from birth to marking were estimated to be higher between birth and marking, than from marking to weaning. Seventy-two percent of producers estimating 2% or less lamb mortality between marking and weaning, as shown in Figure 2b. Producers were requested to identify the methods used to determine the estimated birth to marking mortality rates, with 62% (*n* = 79) using dead lambs observed, 48% (*n* = 61) using scanning to marking figures, and 22% (*n* = 28) using their overall general impression. Among producers, 33% (*n* = 42) identified to use more than one method to estimate lamb mortality between birth and marking. Producers also estimated birth to marking losses by live lamb counts at shearing or weighing lambs at birth. Producers also indicated how losses between marking and weaning were estimated, with 68% (*n* = 88) of respondents using marking to weaning rates, 42% (*n* = 55) used observed dead lambs, and 15% (*n* = 19) using their general impression. Twenty-two percent (*n* = 29) of producers identified to use more than one method to estimate lamb mortality rates from marking to weaning.

Through ordinal logistic regression, no significant associations among farm practices or characteristics and birth to marking rates were found. Marking to weaning rates were significantly related to the farm system (*p* = 0.049), and energy supplementation during mid-pregnancy (*p* = 0.021). Farming system affected estimated mortality rate with sheep-only producers estimating lower marking to weaning mortality (odds ratio 0.273, 95% CI 0.098 to 0.763) than sheep and cropping producers (Wald χ^2^ (1) = 6.135, *p* = 0.013). The odds ratio of producers who supplement ewes during mid-pregnancy with energy and protein of estimating lower marking to weaning mortality was 0.424 (95% CI, 0.203 to 0.886) times that of producers who do not supplement with energy and protein mid-pregnancy (χ^2^ (1) = 5.207, *p* = 0.022). All other farm practices had no significant association with marking to weaning rates.

### 3.5. Energy and Protein Supplementation

Many producers responded that they supplemented ewes with energy and/or protein (94.7%, *n* = 125). Producers also advised on when these energy and protein supplements were given to reproducing ewes (Table 7).

Producers who gave energy and protein supplements identified the supplement provided, with 63% (*n* = 76) supplementing barley, 37% (*n* = 45) supplementing oats and 37% (*n* = 45) supplementing cereal hay. Other less common supplements used by less than 30% of producers included lupins, wheat, Lucerne hay, pre-purchased pellets, and corn. The supplementation amount given per day varied, with 16% (*n* = 18) of producers supplementing less than 200g, 35% (*n* = 40) supplementing 200–399 g, 22% and (*n* = 25) supplementing 400–599 g. Less common supplementation amounts by producers included those supplementing more than 600 g.

### 3.6. Mineral Supplementation

Mineral supplementation was used by 86% (*n* = 113) of respondents. Supplementation of minerals around lambing was provided by 55% (*n* = 72) of producers every year. Supplementation was also provided by less than 15% of producers when ewe body condition score was below target, on certain pasture types, and in poor seasons only. Sixty-two of the 72 producers who supplemented every year identified their reasoning with, 27% (*n* = 17) doing so due to poor pasture quality or deficiencies in pasture, 21% (*n* = 13) for ewe health and 12% (*n* = 8) to provide calcium for lambing ewes. Other less common practices by fewer producers (<10%) for supplementing every year included always offering minerals year-round, providing essential minerals in diet, reducing incidence of metabolic disorders, increasing lamb survival, and milking ability of ewes.

Supplementation type was identified by producers, with 61% (*n* = 69) using pre-purchased licks or blocks, 60% (*n* = 68) supplementing salt, and 58% (*n* = 66) supplementing lime. Other less common supplements used by producers included magnesium, copper sulphate, and selenium. The most common times for producers to supplement ewes was a month before lambing, with 77% of producers (*n* = 87) and during lambing by 66% of producers. Less common times for supplementation included two weeks prior to lambing, after lambing, and during mid-pregnancy.

### 3.7. Cost-Effective Supplement

Producers were asked about the reasons which would prevent them from using a cost-effective supplement to increase lamb survival, with 25% (*n* = 26) noting that nothing would prevent them from using the supplement. The main reasons producers would not use the supplement were identified by 40% (*n* = 42) of producers to be due to time constraints, 31% (*n* = 33) as too wet (paddocks), and 26% (*n* = 28) due to disturbing ewes. Other less common reasons identified by producers for not using the supplement included costs, not bothered to provide a supplement, and not listening to advice.

## 4. Discussion

This survey investigated the practices and perceptions of NSW sheep producers on lamb mortality and supplementation practices around lambing. In recent years, significant research has been undertaken in order to improve lamb mortality rates via supplementation of ewes with various feedstuffs, as perinatal lamb mortality is known to be a major cause of reproductive wastage in sheep breeding enterprises [1]. Previous studies have identified methods to reduce mortality rates [18,19,20]; however, these studies have not determined producer uptake of these strategies and producer knowledge of lamb mortality. Data were collected from 145 respondents in NSW; however, 10,976 businesses were identified in NSW to have breeding ewes [21] and, as such, results should be interpreted with caution and cannot be reliably generalized to the population, although responses were above the targeted number of approximately 100 responses. As per Figure 1, respondents covered most NSW sheep producing areas, providing a good representation of producers in NSW. Some surveys were completed online; therefore, the response rate is unknown as the number of producers reached is unknown. The number of responses could have been affected by surveys being mainly online and the topic being a sensitive area, as producers may not want to disclose some practices. Data may have been biased by producers who are aware of appropriate practices and mortality rates being more likely to complete the survey. Contrastingly, producers who may not consider lamb mortality an issue or having low levels of lamb mortality may cause bias by not taking the time to complete the survey. Consequently, the results may not provide an accurate indication of producer’s knowledge, adoption of supplementation practices, and known mortality rates.

### 4.1. Lamb Mortality

Mortality rates on-farm may be largely underestimated. Nearly half of producers surveyed estimated less than 10% mortality of lambs between birth and marking. Of the producers surveyed, 73% estimated mortality between marking and weaning to be less than 5%, in agreement with previous experimental research that most lamb losses occur within days of birth [1,22]. Although producers only estimated overall lamb mortality in the survey, rather than twin and single loss, this estimate was considerably lower than previously published data. Previously, collected experimental data in Australian merinos found mortality rates of 5–70% of all lambs born, although mortality between 20–25% is more common [1,23,24]. In a NSW lamb survival study, using Merino and Border Leicester X Merino ewes joined to a variety of sires, lamb losses to 3 days post-partum were 11.3% in singles and 20.8% in twins [25]. This mortality rate even in singles to just 3 days post-partum is higher than estimated by almost half of producers in the survey. Furthermore, mortality of 21–38% in twins and 13–21% in singles was noted from data collected on a variety of NSW-based properties from more than 78,000 Merino, 1st Cross or 2nd Cross ewes [24]. These values are considerably higher than most producers estimated in this survey indicating producers may not be aware of the number of lambs being lost between birth and marking, with only 13% of producers estimating more than 20% lamb mortality. Similarly, the reported estimates by producers for lamb mortality are lower than those previously identified by Western Australian sheep producers. Most Western Australian producers estimated lamb survival rates between 70–90%, identifying at least 10–30% lamb mortality [26]. It is, however, possible that lamb mortality estimates by producers were correct, as previously published data are from more than 10 years ago and on-farm practices may have changed. Scanning allows producers to have the opportunity to determine lamb losses by scanning for fetal number and lambing twins and singles separately. Of the producers surveyed between 16–39% of producers scanned but did not scan for fetal number or lamb twins and singles separately. This cohort of producers could increase their knowledge of lamb losses and possibly increase profitability by changing scanning practices to scanning for fetal number and lamb twins and singles separately. If producers are then aware of fetal numbers determining lamb losses in conjunction with ewe losses over the lambing period and the economic effect will allow them to accurately value the costs of strategies to increase lamb survival. Producers may be adopting research which has been undertaken to increase lamb survival especially around nutrition to reduce mortality rates. Research has demonstrated nutrient restriction to ewes can reduce birthweight [27] which is a significant contributor to survival [28]. Following lambing, provision of colostrum and milk is important with ewe nutrition affecting colostrum/milk production [5]. Low colostrum and milk production may cause lamb mortality associated with starvation. Therefore, mortality is likely to be underestimated by producers; although advancements in nutrition may be reducing the on-farm lamb mortality as producers adopt practices to reduce mortalities.

From the data by Fowler [24], 16.5% of single lambs and 31.5% of twin-born lambs died, representing a large number of lambs which producers may not be recognizing as lost lambs based on estimated mortality rate data in the survey. Producers generally used the number of dead lambs observed, scanning to marking rates and their overall general impression to determine mortality from birth to marking. Overall, 76% of producers scan their ewes; however, only 59% of all producers scan for twins and singles with 55% of all producers lambing twins and singles separately. Only 48% of producers used scanning to marking rates to determine lamb mortality when, theoretically, 59% would have the data on fetal numbers available. This represents an area for producers to improve production and to understand the mortality of their lambs better without extra data recording.

### 4.2. Causes of Lamb Mortality

Understanding of the causes of mortality helps to direct producers on improvements to reduce mortality. Causes of mortality can vary due to breed type [29] with some breeds more likely to have feto-pelvic disproportion resulting in dystocia while other breeds have reduced mothering ability resulting in starvation–mismothering–exposure deaths. Producers estimated similar levels of dystocia among breeds (20% Merino, 19% meat, 21% crossbred ewes), and similar to previous reports by Luff [30] of 17.7% mortality. However, another report of nearly 5000 lambs noted 47% mortality due to dystocia [3] and a review of dystocia identified up to 67% of lamb mortalities are associated with dystocia [31] Merinos are known to have lower incidences of dystocia at 4.1% [32] compared to Dorset’s at 34% [33], selection of breed of ewe and ram offering an opportunity to reduce the incidence of dystocia [31]. Starvation–mismothering–exposure deaths were estimated by producers as similar between ewe breeds (merinos 49%, crossbred 45% and meat 40%). These reported values are between the 30% mortality reported by Geenty, Brien [3] and the 58.2% noted by Luff [30]. Mortality due to starvation is consistently high across studies [34], representing an overall general issue in the sheep industry from a variety of causes such as slow-onset milk production, low mothering ability, poor nutrition, and/or lamb factors.

Predation deaths were estimated by producers to be 18% for merinos, 19% for crossbred, and 21% for meat breeds, which are considerably higher than published data at 7–8% [3,30,34]. Refshauge, Brien [34] found that only 0.12% of lamb mortalities were primary predation deaths, demonstrating that predation is generally a secondary cause of death as predators predate compromised lambs. Large numbers of predation deaths can be associated with poor mothering ability and/or proximity to fox habitat [35] as foxes were noted to be the predator in 85% of cases [34]. Therefore, it seems producers in NSW could be over-estimating mortality rates associated with predation, when these lambs may be compromised with the actual cause of mortality associated with starvation–mismothering–exposure, dystocia, or infection.

Infection deaths were estimated by producers to cause 5–6% of all lamb deaths, similar to previous reports of 1–7.6% lamb mortality associated with infection in Australia [34,36]. Although infection only causes a small percentage of overall lamb deaths, it causes a large economic impact, especially in the cases of large infection outbreaks. Ensuring lambs consume adequate immunoglobulins post-birth is important in reducing infection deaths as twin-born lambs have lower serum immunoglobulin concentrations [37] and higher incidences of infection deaths [38]. Therefore, ensuring adequate colostrum intake may also reduce the incidence of infection deaths.

A limitation of the survey design resulted was not knowing whether producers are undertaking practices such as post-mortem examinations to determine cause of lamb mortality. This would have been beneficial to understand in greater detail how producers are determining mortality. It is a necessity that producers are undertaking accurate post-mortem exams on dead lambs to determine the cause of lamb mortality. If producers are not undertaking post-mortem exams their estimations on the cause of lamb mortality may be inaccurate which may affect strategies undertaken to reduce mortalities.

### 4.3. Vaccination

Clostridial vaccination of ewes and lambs is important as these diseases are largely not treatable, with vaccines aiding in their control [39]. Clostridial vaccination is important as clostridia are found naturally in the environment, particularly in soil [40]. Of the producers surveyed, 79% of Merino ewe producers, 80% crossbred ewe producers and 90% of meat ewe producers gave clostridial vaccinations pre-lambing. Clostridial disease vaccination pre-lambing is important as the only way to prevent newborn lambs from clostridial diseases is by ensuring ewes have high circulating antibodies pre-lambing to ensure colostrum is concentrated with immunoglobulins to allow passive transfer to lambs [41]. The ewes require an annual booster 3–4 weeks before lambing because the ewe concentrates antibodies into colostrum in the final 13 days of pregnancy [39,40]. Mortality rates of lambs from clostridial diseases varies greatly, but in outbreaks mortality may reach 30% [40] Vaccination of ewes pre-lambing and lambs at marking is important as clostridial diseases such as navel ill affects newborn lambs and enterotoxaemia affects 4-10 week old lambs and finisher lambs [40], indicating the importance of vaccinating ewes pre-lambing and lambs to ensure production is not impaired. Ninety-six percent of surveyed producers vaccinated lambs, with 99% of these giving a clostridial vaccination. For complete vaccination with an inactivated vaccine such as the clostridial immunizations, two doses 4–6 weeks apart are required [39]. In sheep enterprises, this generally occurs at marking and weaning. Of the producers who vaccinated, 17% of Merino producers and 23% of crossbred lamb producers only vaccinated lambs once, either at marking or weaning which may reduce the effectiveness of the immunization. Some producers identified this was due to lambs being sold soon after vaccination is recommended so they did not administer the booster vaccine as they were not keeping the lambs. However, lambs can be affected by clostridial diseases prior to weaning [40], so producers selling lambs at weaning could still find benefits of vaccination. Although no associations between vaccination and lamb mortality were seen, probably due to the high numbers of producers vaccinating and clostridial diseases occurring sporadically, administering the booster vaccine may not have benefits until after lambs are sold. Without the booster lambs are not immune and may succumb to these diseases prior to slaughter or have a discount applied at slaughter affecting profitability.

The Ovine Johne’s vaccination was the second most common vaccination administered to lambs by 48% of Merino lamb producers and 17% of crossbred lamb producers. Ovine Johne’s Disease is a bacterial infection affecting sheep causing wasting, leading to reproductive wastage and death. With less than 50% of surveyed producers vaccinating, this disease may be having a greater impact on production in some flocks where vaccines are not administered. The *Erysipelas* vaccine controls bacterial arthritis in lambs, and the scabby mouth vaccination is used to control the viral infection causing pustules on the mouth which is most commonly seen in lambs and may reduce production. Vaccination rates for *Erysipelas* and scabby mouth are low with between 14–23 % of producers using these vaccines indicating there may be large numbers of flocks with lost production associated with scabby mouth and *Erysipelas*. Therefore, further extension activities are required to give producers knowledge of cost-effective and appropriate vaccines, vaccination timelines, and to ensure complete vaccination of lambs.

### 4.4. Supplementation

Management choices throughout pregnancy and lactation can affect the mortality rates of lambs, allowing producers to make decisions to improve the survival of lambs. Supplementation of energy and protein to ewes during pregnancy and lactation is a significant factor that can have major implications on marking rates. Supplementation of ewes has been widely studied over recent decades. Undernutrition can have major impacts on the growth of the lamb and its birth weight. In the first 90 days of gestation nutrition affects placental growth allowing lambs to grow to their potential in the last 60 days of gestation [42]. Producers who supplemented ewes during mid-gestation estimated lower marking to weaning mortality rates likely to be a result of producers being more aware of the requirements of the ewe for placental growth, supplementing when conditions are poor, or body condition is low leading to more vigorous lambs through until weaning. Body condition score maintenance was the major reason to supplement with energy and protein as it is associated with increased lamb survival [43]. However, many producers gave supplementation of energy and protein every year regardless of season or body condition maintenance with estimated mortality rates similar. Supplementation following lactation was not included in the survey data and is a limitation as this could affect subsequent production. Without understanding of actual feed availability and supplement amount it is difficult to determine if the supplement was required and the impacts of the supplement. This was a limitation of the survey design; inclusion of the feed availability may have allowed more conclusions to be drawn on supplementation. Previous work noted supplementing ewes with barley as an energy and starch source when pasture availability is high does not result in production benefits [44]. Therefore, these producers may be supplementing ewes when conditions are favorable leading to reduced profit and similar production rates for unsupplemented ewes. Consequently, it may be important for producers to estimate pasture availability to determine if supplementation is beneficial or not. It is important for producers to be aware of production effects associated with supplementation to ensure the desired outcome without over supplementing which may reduce profit margins.

Minerals such as calcium and magnesium are required for fetal and neonatal development and normal physiological functioning of adult sheep. As a result, the demands of the ewe for calcium and magnesium during pregnancy and lactation are high. Eighty-six percent of producers surveyed supplemented minerals around pregnancy and/or lactation with calcium and magnesium being the major supplements provided mainly in the form of loose licks and blocks. Calcium and magnesium supplementation has been shown to improve the energy balance of ewes and improve lamb immune responses [7]. Therefore, most producers are aware of the requirement for minerals and are supplementing ewes.

## 5. Conclusions

This survey has identified the current practices and perceptions of sheep producers in NSW around lamb mortalities and management practices during pregnancy and lactation. It was identified that producers have estimated lower mortality rates of lambs between marking and weaning compared to published literature. This may have follow-on impacts, as if mortalities are not well understood by producers, economic losses associated with mortality are also unknown. Consequently, producers may not be able to determine the effectiveness of practices such as supplementation to increase lamb survival. Ensuring producers are aware of the recommended booster clostridial vaccination, pre-lambing ewe vaccinations, and the lamb *Erysipelas* vaccination may reduce lamb mortalities. Therefore, extension services need to be further developed to enable producers to increase on-farm productivity and to reduce lamb mortality.

## Figures and Tables

**Table 1:** Sheep producers (*n* = 145) participating in a NSW survey in 2019 identified lambing time of ewes by breed.

	Autumn (February–May)	Winter/Spring (June–September)	Dual (Autumn and Winter/Spring)	Continuous	Three Lambings Every 2 Years
Merino	29 (27%)	71 (66%)	5 (5%)	2 (2%)	0 (0%)
Meat	5 (24%)	9 (43%)	4 (19%)	2 (10%)	1 (4%)
Crossbred	13 (25%)	25.5 (50%)	9 (18%)	3.5 (7%)	0 (0%)

* Data presented as the number of producers (% of producers within breed).

**Table 2:** Identification of scanning practice (either scanning for multiples or pregnant/not pregnant) and lambing groups (twins and singles, separate or together) for 104 NSW producers participating in a 2019 survey who identified to pregnancy scan ewes pre-lambing by breed.

	Scanning for Multiples—Lambing Twins and Singles Separately	Scanning for Multiples—Lambing Twins and Singles Together	Scan Pregnant or Not Pregnant
Merino	50 (64%)	4 (6%)	24 (30%)
Meat	10 (71%)	1 (7%)	3 (22%)
Crossbred	32 (84%)	1 (3%)	5 (13%)

* Data presented as the number of producers (% of producers within breed).

**Table 3:** Vaccination type administered to ewes pre-lambing by breed from 114 NSW sheep producers who identified to vaccinate in a 2019 NSW survey.

	Clostridium Vaccine	*Erysipelas polyarthritis*	Ovine Johne’s Disease	Scabby Mouth	Leptospirosis	*Campylobacter fetus*
Merino	84 (100%)	22 (27%)	21 (25%)	10 (12%)	1 (1%)	0 (0%)
Meat	19 (100%)	5 (26%)	7 (33%)	3 (16%)	0 (0%)	1 (5%)
Crossbred	41 (100%)	7 (17%)	7 (17%)	3 (7%)	0 (0%)	0 (0%)

* Data presented as the number of producers (% of producers within breed).

**Table 4:** Vaccination type administered to lambs by breed at marking and/or weaning from 129 NSW sheep producers who identified to vaccinate lambs in a 2019 survey.

	Clostridium Vaccine	*Erysipelas polyarthritis*	Ovine Johne’s Disease	Scabby Mouth	Leptospirosis
Merino	88 (99%)	15 (18%)	40 (48%) ^1^	19 (23%)	1 (1%)
Crossbred	96 (99%)	13 (14%)	16 (17%) ^2^	20 (22%)	1 (1%)

* Data presented as the number of producers (% of producers within breed). ^1^ Three producers only gave Ovine Johne’s vaccine to ewe lambs. ^2^ One producer only gave Ovine Johne’s vaccine to ewe lambs and 2 producers gave Ovine Johne’s vaccine to purebred meat lambs only.

**Table 5:** Lamb marking rates (number of lambs weaned per 100 ewes joined) in good and bad years for Merino, Purebred Meat Breed, and Crossbred ewes as identified by NSW 137 sheep producers.

	Number of Responders	Less Than 49%	50–69%	70–89%	90–99%	100–119%	120–149%	More Than 150%
Merino ewes—a good year	99	0%	0%	8%	19%	47%	23%	3%
Merino ewes—a bad year	96	3%	10%	21%	25%	30%	10%	1%
Meat ewes—a good year	21	0%	0%	0%	5%	24%	33%	38%
Meat ewes—a bad year	20	0%	0%	10%	25%	10%	50%	5%
Crossbred ewes—a good year	51	0%	0%	0%	0%	22%	61%	17%
Crossbred ewes—a bad year	50	0%	2%	6%	10%	40%	38%	4%

* Data presented as the % of producers within breed by good versus bad year. Good year identified as having high feed availability and bad year identified as having low feed availability.

**Table 6:** Causes of lamb mortality and their estimated prevalence identified by 130 producers in a 2019 NSW survey to be responsible for lamb losses by ewe breed.

	Range of Responses (%)	Mean (%)	Median (%)	95% CI (%)
Merino Ewes (*n* = 94)				
Dystocia	0–90	20	10	16–25
Starvation–mismothering–exposure	0–96	49	50	44–54
Predation	0–98	18	10	14–22
Infection	0–30	6	5	4–7
Other	0–90	5	0	3–7
Meat Ewes (*n* = 19)				
Dystocia	0–80	19	10	10–30
Starvation–mismothering–exposure	0–80	40	40	29–51
Predation	0–90	21	20	11–31
Infection	0–20	6	5	3–9
Other	0–100	15	0	3–29
Crossbred Ewes (*n* = 49)				
Dystocia	0–80	21	15	15–27
Starvation–mismothering–exposure	5–100	46	50	38–53
Predation	0–90	19	10	14–25
Infection	0–30	5	1	3–7
Other	0–90	10	0	4–16

* Data presented as the % of producers within breed.

**Table 7:** A 2019 survey of 121 NSW sheep producers timing of energy and protein supplementation under various conditions and production phases around pregnancy/lambing.

	Every Year	Poor Season	Low Body Condition Score
Mid-pregnancy	19 (16%)	7 (6%)	30 (25%)
Month before lambing	24 (20%)	22 (18%)	50 (41%)
2 weeks before lambing	13 (10%)	8 (7%)	27 (22%)
During lambing	16 (13%)	15 (12%)	33 (27%)
After lambing	14 (12%)	14 (12%)	30 (25%)

* Data presented as the number of producers (% of producers within breed). Producers identifying as every year provide the supplement to ewes regardless of weather or seasonal conditions. Poor season supplementing producers provide the supplement only when feed availability is limiting. Producers supplementing under low body condition score supplement when the ewe’s condition is determined by the producer to be low for production.

## Supplementary Materials

The following are available online at https://www.mdpi.com/2076-2615/10/9/1586/s1, File S1, A copy of the ewe lamb producer survey.
