# Supplementary material for: A Survey of New South Wales Sheep Producer Practices and Perceptions on Lamb Mortality and Ewe Supplementation
Source: Animals (Basel). 2020 Sep 5;10(9):1586. doi: 10.3390/ani10091586 (PMC7552230; doi:10.3390/ani10091586)
Supplement: Supplementary file 1 [file animals-10-01586-s001.pdf]

Questionnaire on lamb survival, vaccination and lambing supplementation

1. What type of farming system do you run?<br>Select one only.	□ Sheep ONLY<br>□ Sheep & Cropping<br>□ Sheep & Cattle<br>□ Sheep & Cattle & Cropping<br>□ Other: Please specify: ________________________
2. What breed and number of breeding age ewes do you have?<br>Select up to 3 breeds.<br>Please note which breed you choose for 1,2,3 for future questions.	□ Ewe Breed 1: Breed:__________ No. ewes:________<br>□ Ewe Breed 2: Breed:__________ No. ewes:________<br>□ Ewe Breed 3: Breed:__________ No. ewes:________
3. When do you lamb your ewes?<br>Select all that apply for each breed	Ewe Breed 1:<br>□ Autumn Lambing (March to May)<br>□ Winter/Spring Lambing (June – November)<br>□ Continuous Lambing (Leave rams in all year)<br>□ Other: Please specify: ________________________<br><br>Ewe Breed 2:<br>□ Autumn Lambing (March to May)<br>□ Winter/Spring Lambing (June – November)<br>□ Continuous Lambing (Leave rams in all year)<br>□ Other: Please specify: ________________________<br><br>Ewe Breed 3:<br>□ Autumn Lambing (March to May)<br>□ Winter/Spring Lambing (June – November)<br>□ Continuous Lambing (Leave rams in all year)<br>□ Other: Please specify: ________________________
4. What is the postcode of the farm?	______________________________

5. What is your gender?

□ Male
□ Female
□ Other
□ Rather not say

6. Please state your age?

____

7. Please state how many years have you been farming?

____

8. Please state how many generations has your family been farming?

____

9. Do you vaccinate ewes pre – lambing?
Select all that apply for each ewe breed

Ewe Breed 1:

- □ No
- □ Yes: Which vaccine do you use?
  - □ Glanvac 3
  - □ Glanvac 3S
  - □ Glanvac 3 B12
  - □ Glanvac 3S B12
  - □ Glanvac 6
  - □ Glanvac 6S
  - □ Glanvac 6 B12
  - □ Glanvac 6S B12
  - □ Ultravac 5 in 1
  - □ Cydectin Eweguard
  - □ Cydectin Eweguard SE B12
  - □ Websters 6 in 1
  - □ Websters 6 in 1 B12
  - □ Eryvac
  - □ Gudair
  - □ Leptoshield
  - □ Scabiguard
  - □ Other: Please specify: ___________________

Ewe Breed 2:

- □ No
- □ Yes: Which vaccine do you use?
  - □ Glanvac 3
  - □ Glanvac 3S
  - □ Glanvac 3 B12
  - □ Glanvac 3S B12
  - □ Glanvac 6
  - □ Glanvac 6S
  - □ Glanvac 6 B12
  - □ Glanvac 6S B12
  - □ Ultravac 5 in 1
  - □ Cydectin Eweguard
  - □ Cydectin Eweguard SE B12
  - □ Websters 6 in 1
  - □ Websters 6 in 1 B12
  - □ Eryvac
  - □ Gudair
  - □ Leptoshield
  - □ Scabiguard
  - □ Other: Please specify: ___________________

Ewe Breed 3:

- □ No
- □ Yes: Which vaccine do you use?
  - □ Glanvac 3
  - □ Glanvac 3S
  - □ Glanvac 3 B12
  - □ Glanvac 3S B12
  - □ Glanvac 6
  - □ Glanvac 6S
  - □ Glanvac 6 B12
  - □ Glanvac 6S B12
  - □ Ultravac 5 in 1
  - □ Cydectin Eweguard
  - □ Cydectin Eweguard SE B12
  - □ Websters 6 in 1
  - □ Websters 6 in 1 B12
  - □ Eryvac
  - □ Gudair
  - □ Leptoshield
  - □ Scabiguard
  - □ Other: Please specify: ___________________

10. Do you give any vaccinations to lambs?
Select all that apply for each lamb breed

Merino Lambs
- ☐ Do not have Merino Lambs
- ☐ No
- ☐ Yes: Which vaccine do you use?
  - ☐ Glanvac 3
  - ☐ Glanvac 3S
  - ☐ Glanvac 3 B12
  - ☐ Glanvac 3S B12
  - ☐ Glanvac 6
  - ☐ Glanvac 6S
  - ☐ Glanvac 6 B12
  - ☐ Glanvac 6S B12
  - ☐ Ultravac 5 in 1
  - ☐ Cydectin Weanerguard
  - ☐ Cydectin Weanerguard SE B12
  - ☐ Websters 6 in 1
  - ☐ Websters 6 in 1 B12
  - ☐ Eryvac
  - ☐ Gudair
  - ☐ Leptoshield
  - ☐ Scabiguard
  - ☐ Other: Please specify: ___________________

Meat Breed or Crossbred Lambs
- ☐ Do not have Meat Breed or Crossbred Lambs
- ☐ No
- ☐ Yes: Which vaccine do you use?
  - ☐ Glanvac 3
  - ☐ Glanvac 3S
  - ☐ Glanvac 3 B12
  - ☐ Glanvac 3S B12
  - ☐ Glanvac 6
  - ☐ Glanvac 6S
  - ☐ Glanvac 6 B12
  - ☐ Glanvac 6S B12
  - ☐ Ultravac 5 in 1
  - ☐ Cydectin Weanerguard
  - ☐ Cydectin Weanerguard SE B12
  - ☐ Websters 6 in 1
  - ☐ Websters 6 in 1 B12
  - ☐ Eryvac
  - ☐ Gudair
  - ☐ Leptoshield
  - ☐ Scabiguard
  - ☐ Other: Please specify: ___________________

11. When do you vaccinate lambs?
Select one for each lamb breed

Merino Lambs:

- □ Do not have Merino Lambs
- □ No Vaccination
- □ Marking Only
- □ Weaning Only
- □ Marking and Weaning
- □ Other: Please specify: ___________________________

Meat Breed or Crossbred Lambs:

- □ Do not have Meat Breed or Crossbred Lambs
- □ No Vaccination
- □ Marking Only
- □ Weaning Only
- □ Marking and Weaning
- □ Other: Please specify: ___________________________

12a. If you do not vaccinate lambs or ewes pre-lambing why do you choose not to vaccinate?
Select all that apply

Ewes:

- □ Cost
- □ Time
- □ Not Effective
- □ Other: Please specify:____________________________

Lambs:

- □ Cost
- □ Time
- □ Not Effective
- □ Other: Please specify:____________________________

12b. If you vaccinate your ewes or lambs, why do you choose to vaccinate?
Select all that apply

- □ Have always vaccinated
- □Advised to vaccinate: Please specify who advised you:
  - □ Vet
  - □ Nutritionist
  - □ Family Member
  - □ Rural Store (e.g. Elders/Landmark)
- □ Increase immunity levels
- □ Increase survival of lambs
- □ Other: Please specify: __________________________________

13. Do you scan your ewes?
Select one for each ewe type

Ewe Breed 1:
- □ Twin and single identify and lamb separate
- □ Twin and single identify and lamb together
- □ Scan for pregnant and non-pregnant
- □ Do not scan

Ewe Breed 2:
- □ Twin and single identify and lamb separate
- □ Twin and single identify and lamb together
- □ Scan for pregnant and non-pregnant
- □ Do not scan

Ewe Breed 3:
- □ Twin and single identify and lamb separate
- □ Twin and single identify and lamb together
- □ Scan for pregnant and non-pregnant
- □ Do not scan

Any Comments:___________________________________
____
____

14. What is your average marking percentage **<u>of ewes joined</u>**?
Select one for each type of year for each breed.

Ewe Breed 1:

Good Year (High feed):
- □ More than 150%
- □ 120-149%
- □ 100-119%
- □ 90 – 99%
- □ 70 – 89%
- □ 50 – 69%
- □ Less than 49%

Drought Year (Low Feed):
- □ More than 150%
- □ 120-149%
- □ 100-119%
- □ 90 – 99%
- □ 70 – 89%
- □ 50 – 69%
- □ Less than 49%

Ewe Breed 2:

Good Year (High feed):
- □ More than 150%
- □ 120-149%
- □ 100-119%
- □ 90 – 99%
- □ 70 – 89%
- □ 50 – 69%
- □ Less than 49%

Drought Year (Low Feed):
- □ More than 150%
- □ 120-149%
- □ 100-119%
- □ 90 – 99%
- □ 70 – 89%
- □ 50 – 69%
- □ Less than 49%

Ewe Breed 3:

Good Year (High feed):	Drought Year (Low Feed):
□ More than 150%	□ More than 150%
□ 120-149%	□ 120-149%
□ 100-119%	□ 100-119%
□ 90 – 99%	□ 90 – 99%
□ 70 – 89%	□ 70 – 89%
□ 50 – 69%	□ 50 – 69%
□ Less than 49%	□ Less than 49%

15a. In the past 5 years what percentage of lambs on average die between lamb **birth and marking** do you think**?**
Select one only

□ 0%
□ 1 – 4%
□ 5 – 9%
□ 10 – 14%
□ 15 – 19%
□ 20 – 29%
□ More than 30%
□ Unknown

15b. On what basis do you think this?
Select all that apply

□ Scanning to marking figures
□ Dead lambs observed
□ General Impression
□ Other: Please State: _________________________________

16a. In the past 5 years what percentage of lambs on average die between lamb **marking and weaning** do you think**?**
Select one only

□ 0%
□ 1 – 2%
□ 3 – 4%
□ 5 – 9%
□ More than 10%

16b. On what basis do you think this?
Select all that apply

□ Marking to weaning figures
□ Dead lambs observed
□ General Impression
□ Other: Please State: _________________________________

17. For each ewe breed, what percentage do you think each cause contributes to your lamb losses? Please fill out for up to 3 ewe breeds. **Please ensure the total adds to 100%.**

Breed	Dystocia	Starvation/ Mismothering/ Exposure	Predation	Infection	Other	Total
Example Breed	20%	20%	20%	20%	20%	100%

18a. When do you supplementary feed pre-lambing ewes for energy or protein?
Select one only

□ Every Year
□ Only in Poor seasons
□ When ewe condition is below target
□ Never (Go to Question 19)

18b. If you supplementary feed ewes every year for protein/energy, why do you do this?

____

____

____

18c. What supplements would you typically provide for energy/protein supplementation?
Select all that apply

□ Barley
□ Oats
□ Wheat
□ Lupins
□ Canola Meal
□ Soyabean Meal
□ Lucerne Hay
□ Cereal Hay
□ Other: Please specify:______________________

18d. When do you typically provide the protein/energy supplement to ewes?
Select all that apply

□ During mid pregnancy
□ A month before lambing
□ 2 weeks before lambing
□ During lambing
□ After lambing until weaning

18e. How much per head per day would you typically during lambing feed of the energy/protein supplement?
Select one only

□ Less than 200g
□ 200 – 399g
□ 400 – 599g
□ 600 – 799g
□ 800g – 999g
□ More than 1kg.

19a. When you supplementary feed pre-lambing ewes with minerals?
Select one only

□ Every Year
□ Only in Poor seasons
□ When ewe condition is below target
□ Certain pasture types
□ Never (Go to Question 20)

19b. If you supplementary feed ewes every year with minerals, why do you do this?

____
____
____

19c. What supplements would you typically provide for mineral supplementation?
Select all that apply

□ Purchased Pre-lambing Lick or Block
□ Lime
□ Magnesium
□ Salt
□ Other: Please specify: ____________________________________

19d. When do you typically provide the mineral supplement to ewes?
Select all that apply

□ During mid pregnancy
□ A month before lambing
□ 2 weeks before lambing
□ During lambing
□ After lambing until weaning

20. If you were advised a supplement could be given to ewes pre-lambing, which was cost-effective, what would cause you **not** to use the supplement in a year when pasture is adequate?
Select all that apply

□ Time
□ Possibility of disturbing ewes
□ Too wet
□ Other: Please specify: _____________________________________

Thank you for your assistance in completing this survey.

Please note: By returning this survey you are consenting to the use of the information provided. Raw data sheets and the questionnaire will be stored for 5 years and then destroyed. Strict confidentiality will be maintained.
